# Another Piece of the Obesity–Environment Puzzle: Potential Link between Inflammation and POP-Associated Metabolic Diseases

**DOI:** 10.1289/ehp.120-a164a

**Published:** 2012-04-01

**Authors:** Julia R. Barrett

**Affiliations:** Julia R. Barrett, MS, ELS, a Madison, WI–based science writer and editor, has written for *EHP* since 1996. She is a member of the National Association of Science Writers and the Board of Editors in the Life Sciences.

Epidemiologic research suggests that persistent organic pollutants (POPs) in the environment may contribute to the development of obesity and features of metabolic diseases, such as elevated triglyceride levels, glucose intolerance, and cardiovascular disease. POPs include dioxins and furans—with 2,3,7,8-tetrachlorodibenzo-*p*-dioxin (TCDD) being the most potent of these—and polychlorinated biphenyls (PCBs). Due to their lipophilic nature, these compounds accumulate in adipose (fat) tissue, the site of many endocrine and metabolic functions that are disrupted in metabolic diseases and obesity. A new study outlines a mechanism by which inflammation could play a central role in POP-associated metabolic disease **[*EHP* 120(4):508–514; Kim et al.]**.

TCDD influences genes underlying various cellular processes by binding the aryl hydrocarbon receptor (AhR) and has been shown to stimulate production of enzymes involved in the metabolism of environmental agents. Other halogenated dioxins and furans, as well as coplanar PCBs, may trigger similar effects.

Inflammation, one of the processes affected by these chemicals, is a significant factor in many diseases. Chronic low-grade inflammation in adipose tissue alters its biological function, potentially leading to insulin resistance and other health problems.

In this study, researchers treated both immature precursor cells and mature adipocytes (fat cells) that developed from them with TCDD, dioxin-like PCB126, and non–dioxin-like PCB153. For the *in vivo* portion of the study, wild-type and AhR knockout mice were injected with a single dose of TCDD or corn oil (control).

Mouse adipose tissue was examined after treatment to determine the size of the adipocytes and the presence of macrophages (an indication of inflammation). Gene expression analysis determined the numbers and types of up- and downregulated genes in both cultured cells and adipose tissue, with those involved in inflammation being the most significantly upregulated. However, the PCB153-associated gene regulation pattern was unlike the patterns for PCB126 and TCDD, whose effects could be blocked by an AhR antagonist *in vitro*.

The inflammatory response was strongly induced in precursor cells and to a lesser degree in adipocytes, and this response was mediated through the AhR. The findings were confirmed in the rodent tissue and are congruent with epidemiologic studies. Caution is required in extending the findings to human health, however, because the rodent exposure regimen was dissimilar from typical human exposures. Nevertheless, this study highlights what may prove to be one of the major mechanisms by which POPs affect disease development.

